# Distinct oral-associated gastric microbiota and *Helicobacter pylori* communities for spatial microbial heterogeneity in gastric cancer

**DOI:** 10.1128/msystems.00089-24

**Published:** 2024-06-28

**Authors:** Lei Lei, Lin-Yong Zhao, Ran Cheng, Hongyu Zhang, Mengying Xia, Xiao-Long Chen, Valentin Kudriashov, Kai Liu, Wei-Han Zhang, Han Jiang, Yi Chen, Liang Zhu, Hongmei Zhou, Kun Yang, Tao Hu, Jian-Kun Hu

**Affiliations:** 1 State Key Laboratory of Oral Diseases and National Center for Stomatology and National Clinical Research Center for Oral Diseases and Frontier Innovation Center for Dental Medicine Plus, West China Hospital of Stomatology, Sichuan University, Chengdu, China; 2 Gastric Cancer Center and Laboratory of Gastric Cancer, State Key Laboratory of Biotherapy, West China Hospital, Sichuan University, and Collaborative Innovation Center for Biotherapy, Chengdu, China; 3 Division of Gastrointestinal Surgery, Department of General Surgery, West China Hospital, Sichuan University, Chengdu, China; 4 Department of Infection Control, West China Hospital, Sichuan University, Chengdu, China; 5 Nuffield Department of Medicine, University of Oxford, Target Discovery Institute, Center for Medicines Discovery, Oxford, United Kingdom; 6 Nuffield Department of Medicine, University of Oxford, Chinese Academy of Medical Sciences (CAMS), CAMS Oxford Institute (COI), Oxford, United Kingdom; Istanbul Medipol University School of Medicine, Istanbul, Turkey

**Keywords:** gastric cancer, microbiota, *Helicobacter pylori*, oral-associated gastric microbiota, overall survival

## Abstract

The gastric microbial community plays a fundamental role in gastric cancer (GC), and the two main anatomical subtypes of GC, non-cardia and cardia GC, are associated with different risk factors (*Helicobacter pylori* for non-cardia GC). To decipher the different microbial spatial communities of GC, we performed a multicenter retrospective analysis to characterize the gastric microbiota in 223 GC patients, including *H. pylori*-positive or -negative patients, with tumors and paired adjacent normal tissues, using third-generation sequencing. In the independent validation cohort, both dental plaque and GC tumoral tissue samples were collected and sequenced. The prevalence of *H. pylori* and oral-associated bacteria was verified using fluorescence *in situ* hybridization (FISH) assays in GC tumoral tissues and matched nontumoral tissues. We found that the vertical distribution of the gastric microbiota, at the upper, middle, and lower third sites of GC, was likely an important factor causing microbial diversity in GC tumor tissues. The oral-associated microbiota cluster, which included *Veillonella parvula*, *Streptococcus oralis*, and *Prevotella intermedia*, was more abundant in the upper third of the GC. However, *H. pylori* was more abundant in the lower third of the GC and exhibited a significantly high degree of microbial correlation. The oral-associated microbiota module was co-exclusive with *H. pylori* in the lower third site of the GC tumoral tissue. Importantly, *H. pylori*-negative GC patients with oral-associated gastric microbiota showed worse overall survival, while the increase in microbial abundance in *H. pylori*-positive GC patients showed no difference in overall survival. The prevalence of *V. parvula* in both the dental plaque and GC tissue samples was concordant in the independent validation phase. We showed that the oral-associated species *V. parvula* and *S. oralis* were correlated with overall survival. Our study highlights the roles of the oral-associated microbiota in the upper third of the GC. In addition, oral-associated species may serve as noninvasive screening tools for the management of GC and an independent prognostic factor for *H. pylori*-negative GCs.

**IMPORTANCE:**

Our study highlights the roles of the oral-associated microbiota in the upper third of gastric cancer (GC).We showed that the oral-associated species *Veillonella parvula* and *Streptococcus oralis* were correlated with overall survival. In addition, oral-associated species may serve as noninvasive screening tools for the management of GC and an independent prognostic factor for *Helicobacter pylori*-negative GCs.

## INTRODUCTION

Gastric cancer (GC) is the fifth most frequently diagnosed malignant cancer and the fourth leading cause of cancer-related deaths worldwide, with more than one million new cases and approximately 770,000 deaths in 2020 (1, 2). Host-related factors, including genetic predisposition, environmental exposure factors, and microbial infections, contribute to gastric tumorigenesis (3, 4). *Helicobacter pylori* (*H. pylori*) infection plays a crucial role in mucosal inflammation, induces histological changes, and promotes the degradation of the architecture and integrity of the gastric epithelium, which is recognized as an important etiological factor of GC (5, 6). However, only 3% of *H. pylori*-infected individuals develop GC (7). In addition, successful *H. pylori* eradication does not completely prevent the development of GC. In some cases, tumor progression is observed even after *H. pylori* eradication (8). *H. pylori*, along with other non-*H*. *pylori* urease-positive *Staphylococcus epidermidis* and *Streptococcus salivarius*, plays a role in gastric carcinogenesis (9). One study showed that bacterial factors other than *H. pylori* also contribute to gastric tumorigenesis (10).

Regarding the anatomical proportion of GC, two main anatomical subtypes exist, non-cardia and cardia, with different risk factors (11). *H. pylori* infection is regarded as a major cause of non-cardia gastric cancer (NCGC), as several epidemiological studies have consistently shown a strong association between NCGC and *H. pylori* infection (9–11). On the other hand, cardia gastric cancer (CGC) accounts for approximately one-third of all GC cases globally. However, *H. pylori* infection is not associated with the development of CGC, with either a null or reduced risk (12–15). Esophagogastric junctional adenocarcinoma mostly occurs in the cardiac region, and its incidence has increased in recent years (16, 17). The spatial heterogeneity of GC microbial diversity has not been clearly elucidated because of differences in the anatomical proportions of the stomach.

The gastrointestinal tract traverses from the mouth to the anus, and diverse microbial communities specifically survive and reside in individual ecological niches (18). Numerous oral bacteria are swallowed daily, which might be the potential origin of the enriched microbiota in GC (19, 20). The interaction and relationship between *H. pylori* and the oral microbiota in GC and the underlying mechanisms of the oral microbiota in carcinogenesis have been discussed (21). Recent studies have shown that poor oral hygiene is associated with an increased risk of GC (22, 23). Based on gastric samples collected from upper gastrointestinal endoscopy, certain oral species are correlated with pathways associated with inflammation in gastric intestinal metaplasia (24). Microbiome sequencing analysis revealed that enriched oral microbial taxa are positively associated with the risk of GC (25). However, the role of oral bacterial factors interacting with *H. pylori* in the microbial diversity of GC still warrants further investigation.

In the present study, we analyzed the composition and diversity of the gastric microbiota, aiming to (i) reveal the spatial heterogeneity of the microbial diversity of GC associated with clinicopathological features and (ii) construct a co-occurrence network of oral-associated microbes interacting with *H. pylori* in the microbial diversity of GC. Overall, our study revealed the spatial heterogeneity of the microbial network in the GC microbiota and validated the presence of oral-associated microbes in GC tumor tissue and dental plaque. The core oral-associated gastric microbiota may serve as a potential predictive marker and a prevention strategy for GC management.

## RESULTS

### Overview of clinicopathological characteristics and bacterial diversity differences between GC tumoral and matched nontumoral tissues

**Fig 1:**
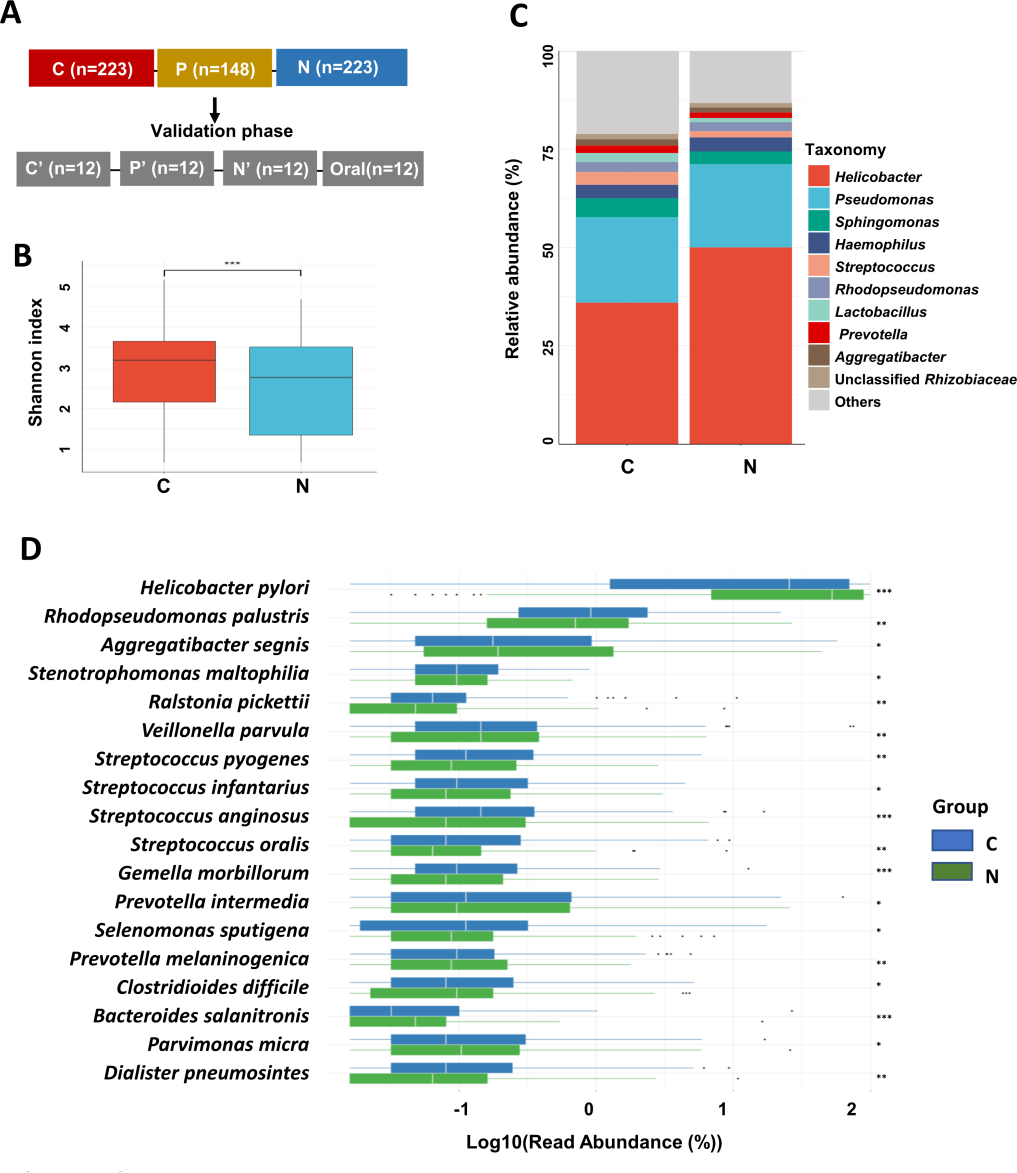
Identification of bacterial taxa important in the entire stomach for GC. (**A**) Study design, C: tumoral tissues; P: peritumoral tissues; N: matched nontumoral tissues. (**B**) The Shannon’s index indicated the alpha-diversity between C group (GC tumoral tissue) and N group (matched nontumoral tissues). (**C**) The top 10 bacterial genus compositions in GC and matched non-tumoral tissues. (**D**) Random Forest Machine learning indicated 18 species differentially in relative abundance between GC tumoral tissues (C group, *n* = 223) and matched nontumoral tissues (N group, *n* = 223, ^*^P<0.05, ^**^P<0.01, and ^***^P<0.001).

The median age of the 223 patients was 60 years (range, 21–85 years), and 157 were male (70.4%). The demographic and clinicopathological characteristics of the 223 participants are shown in Table 1 and Table S1. The bacterial species in the tumor tissues were correlated with demographic and clinicopathological features (Fig. S1). The top 20 most abundant microbial species in GC tumoral tissues (C group) and matched nontumoral tissues (N group) are illustrated in Fig. S2 and Table S2. Figure 1A shows the study design. The alpha diversity (Shannon index) of the gastric microbiota was analyzed using the Kruskal‒Wallis test. Compared with those of the N group, the microbiomes of the C group had significantly greater Shannon-estimated microbial richness (*P* = 0.0002346, Fig. 1B).

**TABLE 1:** Patients and clinicopathologic factors^*a*^

Factors	Microbial diversity			Microbial abundance change			*H. pylori*			Oral species taxon		
	High *n* = 104	Low *n* = 119	*P* value	Increase (*n* = 145)	Decrease (*n* = 78)	*P* value	Positive (*n* = 168)	Negative (*n* = 55)	*P* value	Positive (*n* = 75)	Negative (*n* = 148)	*P* value
Gender (F/M)	28/76	38/81	0.414	41/104	25/53	0.556	51/117	15/40	0.664	21/54	45/103	0.710
Age (65≤/>65 years)	72/32	83/36	0.933	99/46	56/22	0.586	118/50	37/18	0.678	42/33	11/35	<0.001
BMI (24≤/>24 kg/m^2^)	63/41	79/40	0.368	91/54	51/27	0.697	104/64	37/18	0.474	45/30	97/51	0.416
Tumor size (6≤/>6 cm)	69/35	83/36	0.586	94/51	58/20	0.145	115/53	37/18	0.871	45/30	107/41	0.063
Longitudinal location			0.011			0.217			<0.001			0.007
Upper third	46	30		53	23		33	43		36	40	
Middle third	13	20		24	9		24	9		10	23	
Lower third	45	69		68	46		111	3		29	85	
Cross-sectional location			0.770			0.172			0.457			0.698
Lesser curvature	61	74		94	41		101	34		45	90	
Greater curvature	13	15		14	14		23	5		12	16	
Anterior wall	7	10		17	13		22	5		8	19	
Posterior wall	23	20		20	10		22	11		10	23	
Macroscopic type			0.241			0.289			0.611			0.708
Borrmann 0-II	58	57		71	44		85	30		40	75	
BorrmannIII-IV	46	62		74	34		83	25		35	73	
Tumor differentiation			0.171			0.802			0.398			0.258
Well/moderately	49	67		76	39		94	22		43	73	
Poorly/undifferentiated	55	52		69	38		74	33		32	75	
Lauren classification			0.397			0.923			<0.001			0.221
Intestinal	45	57		67	35		93	9		32	70	
Diffused	38	46		55	29		54	30		26	58	
Mixed	21	16		23	14		21	16		17	20	
Her2 (positive/negative)	14/83	22/92	0.349	18/121	18/54	0.027	25/133	11/42	0.409	17/56	19/119	0.080
Chemotherapy (yes/no)	70/34	78/41	0.781	97/48	51/27	0.820	114/54	34/21	0.411	47/28	101/47	0.405
T Stage (T1-2/T3-4)	25/79	22/97	0.311	32/113	15/63	0.620	37/131	10/45	0.544	14/61	33/115	0.530
N Stage (N0-1/N2-3)	42/62	30/89	0.016	41/104	31/47	0.081	51/117	21/34	0.281	25/50	47/101	0.812
M Stage (M0/M1)	92/12	101/18	0.433	129/16	64/14	0.149	147/21	46/9	0.466	65/10	128/20	0.970
TNM stage (*n*)			0.059			0.429			0.576			0.538
I	13	10		14	9		19	4		5	18	
II	31	20		33	18		36	15		20	31	
III	48	71		82	37		92	27		40	79	
IV	12	18		16	14		21	9		10	20	

^*a*^ BMI: body mass index; T stage, N stage, M stage, and TNM stage evaluated according to the seventh edition of the AJCC TNM staging system. The samples with ≤1% *H. pylori* relative abundance were defined as *H. pylori*-negative, while samples with >1% *H. pylori* relative abundance were *H. pylori*-positive as previously described. Similarly, samples with ≤1% relative abundance of species were regarded as negative, while samples with >1% relative abundance of species were regarded as positive. Oral species taxon includes *Veillonella parvula*, *Streptococcus oralis*, *Porphyromonas gingivalis*, and *Prevotella intermedia*. With the optimal Operational Taxonomic Unit (OTU) cutoff value of 207 determined by X-tile using the minimum *P* value from the log-rank *χ*^2^ test, samples were grouped into high and low microbial diversity groups.

**Fig 2:**
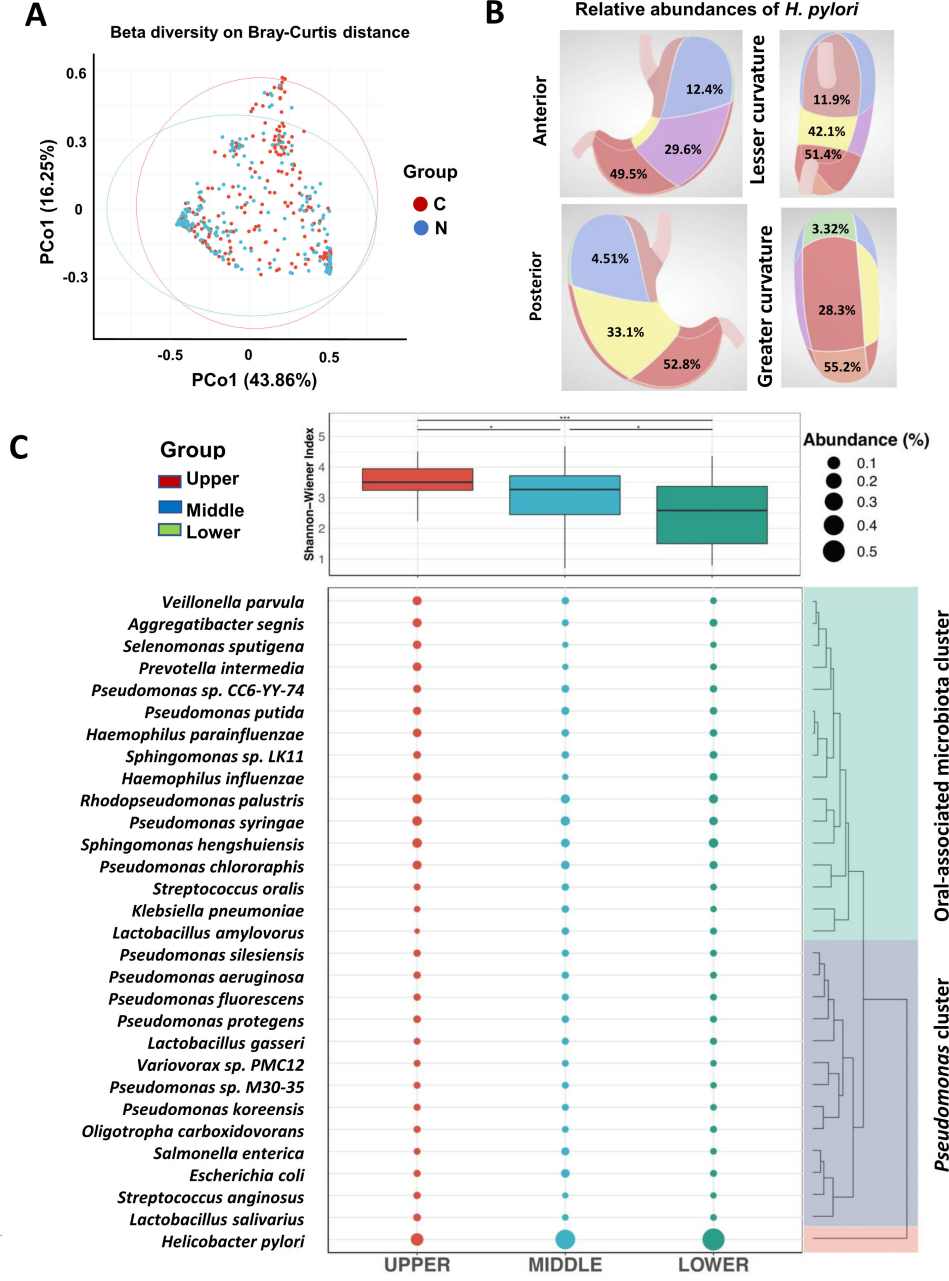
Spatial heterogeneous of microbial diversity in GC. (**A**) Beta diversity based on Bray-Curtis of the GC and matched nontumoral tissues microbial community. (**B**) The average relative abundance of *H. pylori* abundant taxa in each region was calculated: 49.5% in the lower anterior region, 55.2% in the lower greater curvature region, 51.4% in the lower lesser curvature region, 52.8% in the lower posterior region, 29.6% in the middle anterior region, 28.3% in the middle greater curvature region, 42.1% in the middle lesser curvature region, 33.1% in the middle posterior region, 12.4% in the upper anterior region, 3.32% in the upper greater curvature region, 11.9% in the upper lesser curvature region, and 4.51% in the upper posterior region. (**C**) The vertical gradient variations of alpha-diversity and community compositions (*P* < 0.05, ^**^P<0.01, and ^***^P<0.001).

The relative abundances of the top 10 genera in tumoral and matched nontumoral tissues are presented in Fig. 1C. We constructed a random forest classifier model to assess the differential bacterial species between the N and C groups based on their relative abundances (Fig. 1D, *n* = 223). The relative abundance of *H. pylori* was significantly greater in the N group than in the C group (*P* = 0.0001). Interestingly, some of these species are common oral species taxa, including *Veillonella parvula* (*V. parvula*, *P* = 0.0098), *Streptococcus oralis* (*S. oralis*, *P* = 0.0034), *Streptococcus anginosus* (*S. anginosus*, *P* = 0.0006), and *Prevotella intermedia* (*P. intermedia*, *P* = 0.0430), which were enriched in GC tumoral tissues. The permutation test showed that the overall differences in beta diversity according to Bray-Curtis dissimilarity (Fig. 2A) between the C and N groups were significant (*P* = 0.005), indicating that the microbial communities were distinct between the two groups.

### The vertical spatial heterogeneity between bacterial diversity and species richness was distinct, especially for *H. pylori* and oral-associated microbiota

To reduce the complexity to manageable proportions, the gastric anatomical distributions were divided by the vertical and cross-sectional classifications, and the average relative abundance of *H. pylori* was calculated, indicating that *H. pylori* was more abundant in the lower third of the GC (Fig. 2B). The tumoral spatial location was another factor associated with the microbial diversity, which indicated that the alpha diversity (Shannon index using the Kruskal‒Wallis test) of the gastric microbiota was significantly greater at the upper third site with little individual variance than at the middle (*P* = 0.03776) and lower (*P* = 0.0000000001) third sites (Fig. 2C). In addition, based on the clinicopathological characteristics of the tumors, tumor N stage (lymph node status) was identified as a risk factor for microbial diversity (*P* = 0.016; Table 1), which indicated that the greater the microbial diversity, the lower the possibility of lymph node metastasis in GC patients.

According to the relative abundances of the top 30 species, the dotted areas represent the mean relative abundances (Fig. 2C), in which the species are clustered into three major clusters: *H. pylori*, *Pseudomonas*, and oral-associated microbiota. *H. pylori* was more abundant in the lower third site, whereas co-occurring oral-associated microbiota clusters, including *V. parvula*, *S. oralis*, *S. anginosus*, and *P. intermedia*, were more abundant in the upper third of the GC. Gastric samples with ≤1% *H. pylori* relative abundance were defined as *H. pylori*-negative, while gastric samples with >1% *H. pylori* relative abundance were *H. pylori*-positive (10). The clinical characteristics implied 168 patients were *H. pylori*-positive, 111 of whom had GC in the lower third of the stomach (*n* = 111/168, 66.07%; Table 1). In addition, the differences in the alpha diversity (Shannon index) between *H. pylori-*negative and *H. pylori*-positive GC individuals in the upper third site were analyzed (Table S3). Interestingly, the alpha diversity of the gastric microbiota was significantly greater in the matched nontumor tissues of *H. pylori*-negative patients (3.08 ± 0.87) than in those of *H. pylori*-positive patients (2.44 ± 1.14) at the upper third site according to the Kruskal‒Wallis test (*P* = 0.03139). However, the alpha diversities of the gastric microbiota in the tumor tissues of *H. pylori*-positive (3.52 ± 0.49) and *H. pylori*-negative patients (3.43 ± 0.79) in the upper third of the stomach were not significantly different (*P* = 0.730).

**Fig 3:**
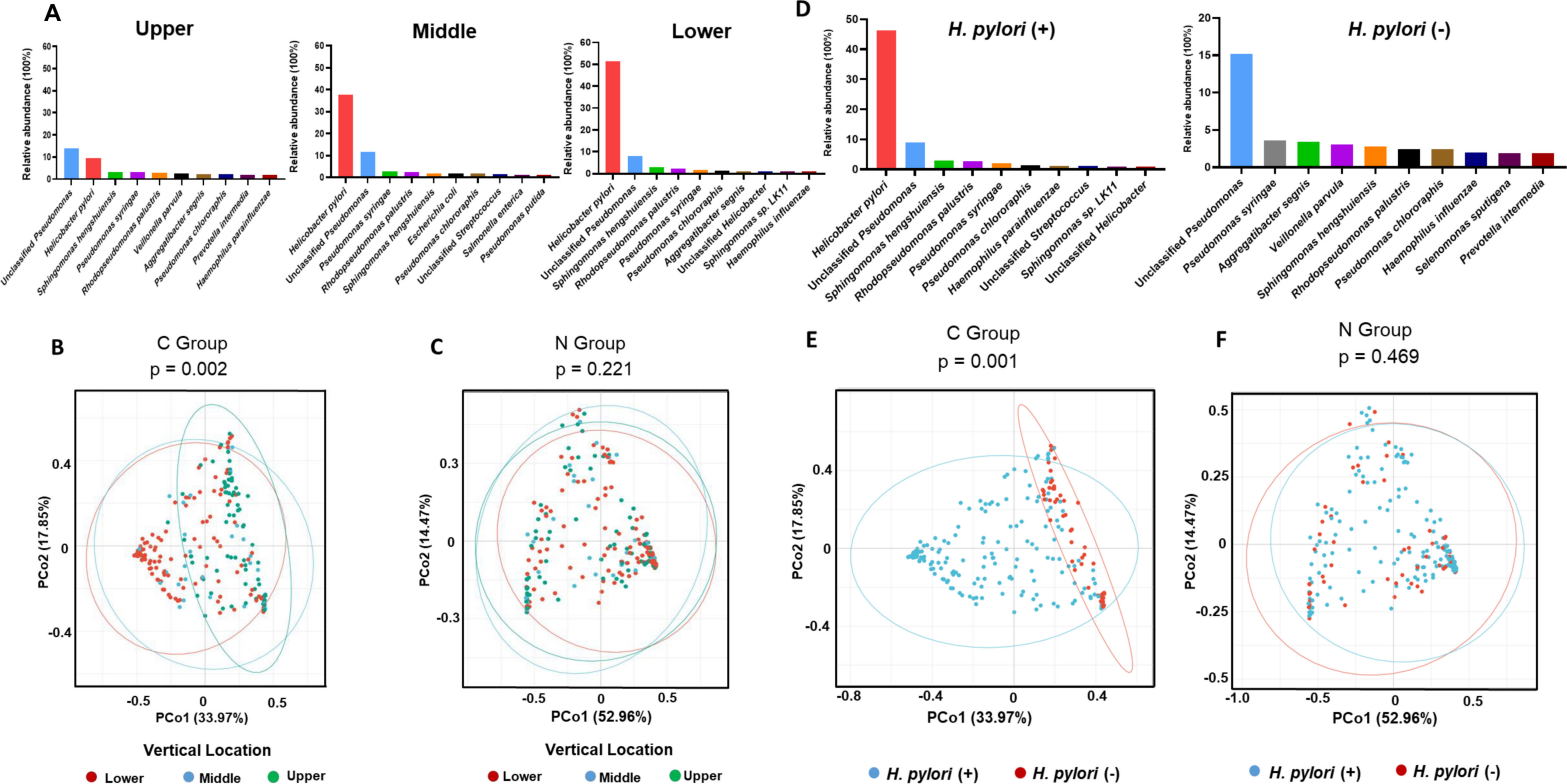
Vertical spatial heterogeneity between bacteria diversity and species richness for *H. pylori*-negative GC. (**A**) The bar graph of the top 10 abundant distinct microbial species for GC in categorized upper (*n* = 76), middle (*n* = 33), and lower (*n* = 114) locations. Beta diversity analysis was performed using Bray-Curtis distance to assay similarities in the GC tumoral (B, *P* = 0.002) or matched nontumoral samples (C, *P* = 0.221) from the upper, middle, and lower third site of GC. (**D**) The bar graph charts of the top 10 abundant microbial species for *H. pylori*-positive GCs (*n* = 168) and *H. pylori*-negative GCs (*n* = 55). (**E**) Beta diversity analysis using Bray-Curtis distance was performed to compare the microbial composition of GC tumoral sample in *H. pylori*-negative tissues (*P* = 0.001). (**F**) Beta diversity analysis using Bray-Curtis distance of matched nontumoral samples in *H. pylori*-negative tissues (*P* = 0.469).

The relative abundances of the top 10 bacterial species in the GCs at the upper, middle, and lower third sites are shown in Fig. 3A. Notably, the mean relative abundances of *H. pylori* in the upper, middle, and lower third sites were 9.4%, 37.6%, and 51.6%, respectively. Regarding the effect of vertical spatial heterogeneity on bacterial community stability, beta diversity significantly differed among the upper, middle, and lower third sites of GC tumoral tissues according to the Bray-Curtis distance (Fig. 3B; *P* = 0.002), whereas the beta diversities of microbial communities among the upper, middle, and lower third sites in matched nontumoral tissues were not significantly different (Fig. 3C; *P* = 0.221). Based on the alpha and beta diversity analysis, we found that gastric vertical distribution was likely an important factor causing microbial variations in the GC tumoral tissues, and the lower third site GC microbiota may have a high degree of dysbiosis, consistent with its reduced bacterial diversity (Fig. 2C).

The relative abundances of the top 10 bacterial species in the tumoral tissues of *H. pylori*-negative GCs or *H. pylori*-positive GCs are presented in Fig. 3D. The Pearson correlation coefficient indicated that the abundance of *H. pylori* was negatively correlated with that of oral bacteria such as *V. parvula*, *P. intermedia*, *S. oralis*, and *S. anginosus* (*r* = −0.220 and *P* = 0.000032564157; Table S2). Interestingly, the overall microbial composition according to the beta diversity with Bray-Curtis distance was significantly different between *H. pylori*-positive and *H. pylori*-negative patients in the GC tumoral tissues (Fig. 3E and *P* = 0.001) but nonsignificant in the matched nontumoral tissues (Fig. 3F and *P* = 0.469). In addition, beta diversity analysis revealed that greater differences in bacterial composition were present within the *H. pylori*-positive GC group (Fig. 3E). To investigate how the presence or absence of *H. pylori* colonization affects the region-specific microbiota, the gastric microbiota was analyzed using the permutation test based on the Bray-Curtis distance (Table S4), which indicated that, in *H. pylori*-positive individuals, the beta diversity of the gastric microbiota at the upper third site was significantly different from that at the middle (*P* = 0.002) or lower (*P* = 0.015) third sites. In contrast, in *H. pylori*-negative individuals, the beta diversity of the gastric microbiota at the upper third site was not significantly different from that at the middle (*P* = 0.297) or lower (*P* = 0.931) third sites. The potential mechanisms by which *H. pylori* affects the vertical spatial heterogeneity of microbial diversity in GC pathogenesis need further exploration.

### The networks reveal different co-occurrence patterns in vertical distributions influenced by oral-associated microbiota clusters and *H. pylori*

**Fig 4:**
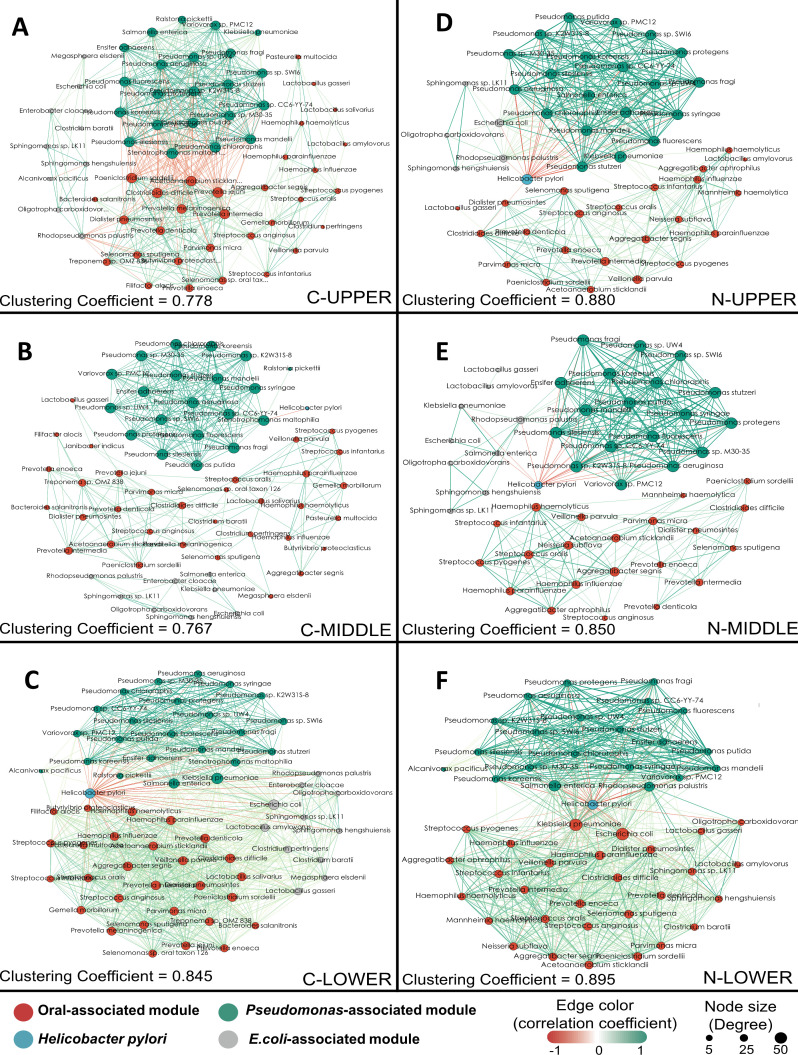
The networks reveal different co-occurrence patterns in vertical distributions induced by oral-associated microbiota cluster and *H. pylori.* (**A**) The upper third site in tumoral tissues (*n* = 76). (**B**) The middle third site in tumoral tissues (*n* = 33). (**C**) The lower third site in tumoral tissues (*n* = 114). (**D**) The upper third site in matched nontumoral tissues (*n* = 76). (**E**) The middle third site in matched nontumoral tissues (*n* = 33). (**F**) The lower third site in matched nontumoral tissues (*n* = 114).

To further visualize the correlations of microbial abundance, we calculated the degrees of Spearman rank correlations of the relative abundance of OTUs in all groups (*P* < 0.001 |*r*| > 0.6). The microbial network was constructed, visualized, and summarized using Gephi (v 0.9.2). The oral-associated microbiota cluster, which is represented by red dots and defined as the “core oral-associated gastric microbiota,” includes *V. parvula*, *P. intermedia*, *S. oralis*, *S. anginosus*, *Haemophilus influenzae*, *Streptococcus pyogenes*, and *Prevotella melaninogenica*. To identify microbial correlations in the gastric community, we adopted co-occurrence patterns of *H. pylori*, *Pseudomonas*, and oral-associated microbiota modules (Fig. 4). The larger the size of each dot, the higher the degree of microbial correlation in the network. Overall, the degree of closeness of the microbial network presented a pattern of “high–low–higher,” both in the GC tumoral tissue and matched nontumoral tissues. The clustering coefficient index revealed that the connectivity of the microbiota network was greater in the upper (0.778 in the C group and 0.88 in the N group) and lower third sites (0.845 in the C group and 0.895 in the N group). Stronger negative correlations were observed between the *Pseudomonas* cluster and oral-associated microbiota cluster (Fig. 4A) in the upper third of the GC tumoral tissues, whereas an uncorrelated tendency was observed in the middle third of the GC tumoral tissues (Fig. 4B). Conversely, the oral microbiota and *Pseudomonas* cluster were more positively correlated via an *H. pylori*-mediated co-exclusion relationship, particularly in the stomach of the lower third site (Fig. 4C and F). In particular, *H. pylori* at the lower third site of the GC exhibited a significantly high degree of microbial correlation in the network, suggesting that *H. pylori* may be involved in an interchange of bacteria in the gastric microbiota.

A phylogenetic tree (Fig. S3) was constructed for the 16S rRNA genes of the core microorganisms in all networks (Fig. 4). A linear discriminant analysis effect size approach to compare different bacteria was applied to the gastric microbiota among GC tumoral tissues, peritumoral tissues, and matched non-tumoral tissues (Fig. S4). In combination with the previous cluster analysis based on community composition, we speculated that there is an obvious quantitative and evolutionary co-occurrence relationship among oral-associated microbiota, which may have special symbiotic relationships to achieve specific functions. The most abundant bacterial species among the oral-associated microbiota module included *V. parvula*, *P. intermedia*, *S. oralis*, *S. anginosus*, *H. influenzae*, *Porphyromonas gingivalis*, and *P. melaninogenica*, which is defined as the “core oral-associated gastric microbiota” in the present study. A 50% occurrence standard was used to identify the core oral microbiome, meaning that any OTU detected in at least 50% of the samples was considered a core member. Additionally, the minimum relative abundance threshold to be considered for core membership was 0.001 (26).

### Differences in bacterial community structures from GC tumoral tissues, peritumoral tissues, and matched nontumoral tissues

**Fig 5:**
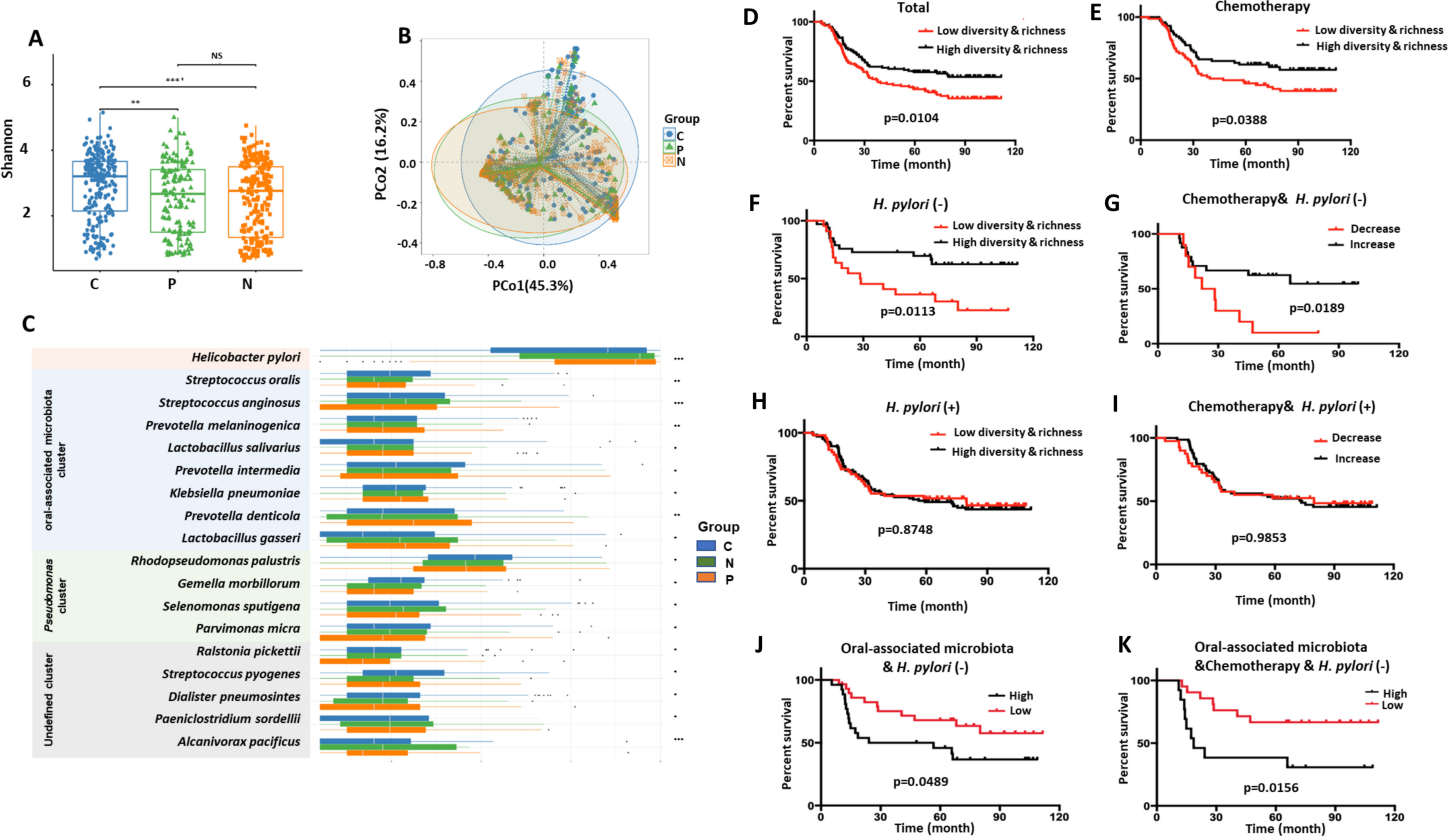
The differences in bacterial community structures from GC tumoral tissues, peritumoral tissues, and matched nontumoral tissues, correlating with the overall survival. (**A**) The differences in the microbial alpha diversity and microbial richness among GC tumoral tissue (C group), peritumoral tissues (P group), and matched nontumoral tissue (N group). (**B**) The similarities and differences were determined by PCoA plots, indicating that the GC tumoral (C group) showed distinct differences from peritumoral tissues (P group) and matched nontumoral (N group) tissues. (**C**) Random Forest Machine learning indicated the enriched species in differential abundance among GC tumoral tissues (*n* = 223), peritumoral tissues (*n* = 148), and matched nontumoral tissues (*n* = 223, ^*^P<0.05, ^**^P<0.01, and ^***^P<0.001). (**D**) The overall survival of GC patients stratified by low and high diversity and richness (*P* = 0.0104). (**E**) The survival analysis of GC patients with adjuvant chemotherapy stratified by low and high diversity and richness (*P* = 0.0388). (**F**) The overall survival of *H. pylori*-negative GC patients stratified by low and high diversity and richness (*P* = 0.0113). (**G**) The survival analysis of *H. pylori*-negative GC patients with adjuvant chemotherapy stratified by low and high diversity and richness (*P* = 0.0189). (**H**) The overall survival of *H. pylori*-positive GC patients stratified by low and high diversity and richness (*P* = 0.8748). (**I**) The survival analysis of *H. pylori*-positive GC patients with adjuvant chemotherapy stratified by low and high diversity and richness (*P* = 0.9853). (**J**) The survival analysis of *H. pylori*-negative GC patients associated with low and high oral microbial taxa (*P* = 0.0489). (**K**) The survival analysis of *H. pylori*-negative GC patients with adjuvant chemotherapy stratified by low and high oral microbial taxa (*P* = 0.0156).

A further notable finding of the present study was that the microbial community profiles in tumor progression, 223 GC tumoral tissues and corresponding matched nontumoral tissues, and 148 peritumoral tissues were compared for additional analysis (Table S2). The alpha diversity of the gastric microbiota was significantly increased in the GC tumoral tissues when compared to the peritumoral and matched nontumoral tissues (Fig. 5A), suggesting that the passenger bacteria in the GC tumoral tissues partially colonized the corresponding matched peritumoral and nontumoral tissues. The GC tumoral tissues showed significantly distinct differences from the matched peritumoral and nontumoral tissues in the beta diversity determined by principal co-ordinates analysis (PCoA) (Fig. 5B).

The most enriched species in the GC tumor tissues was *H. pylori*, which accounted for 35.1% of the relative abundance. Interestingly, the relative abundance of *H. pylori* was 48.9% in the matched nontumoral tissues, which was even greater (Table S5). Using random forest machine learning analyses, significantly differentially abundant taxa showed that *H. pylori* was the most enriched species, and oral-associated microbiota cluster members, including *S. oralis* and *P. intermedia*, were enriched in GC tumor tissues (Fig. 5C). *Rhodopseudomonas palustris* was the second most enriched species, revealing the role of this species in gastric microbiota ecology.

### The core oral-associated gastric microbiota correlates with overall survival, especially in *H. pylori*-negative GCs, and reveals characteristic bacteria consistent with oral and gastric communities

Compared with the low microbial diversity and richness group, the high microbial abundance group had better 5-year overall survival (*P* = 0.0104, hazard ratio (HR) = 1.589, 95% CI: 1.115–2.248; Fig. 5D) and benefited more from chemotherapy (*P* = 0.0388, HR = 1.623, 95% CI: 1.025–2.136; Fig. 5E). Additionally, the increase in microbial abundance in GC tumor tissues was associated with a significant improvement in overall survival (*P* = 0.0113, HR = 2.2528, 95% CI: 1.265–6.010; Fig. 5F) and gained more benefit from chemotherapy (*P* = 0.0189, HR = 2.773, 95% CI:1.251–10.12, Fig. 5G) for GCs without *H. pylori* infection. However, the increase in microbial abundance in *H. pylori*-positive GC patients showed no difference in overall survival (*P* = 0.8748; Fig. 5H) and no benefit from chemotherapy (*P* = 0.9853; Fig. 5I). Especially in *H. pylori*-negative GCs, when compared with oral species-negative GCs, GCs with oral microbial taxa were associated with worse overall survival (Fig. 5J; *P* = 0.0489, HR = 2.120, 95% CI:1.006–4.073), and the patients benefited less from chemotherapy (Fig. 5K; *P* = 0.0156, HR = 3.148, 95% CI:1.298–11.25).

**Fig 6:**
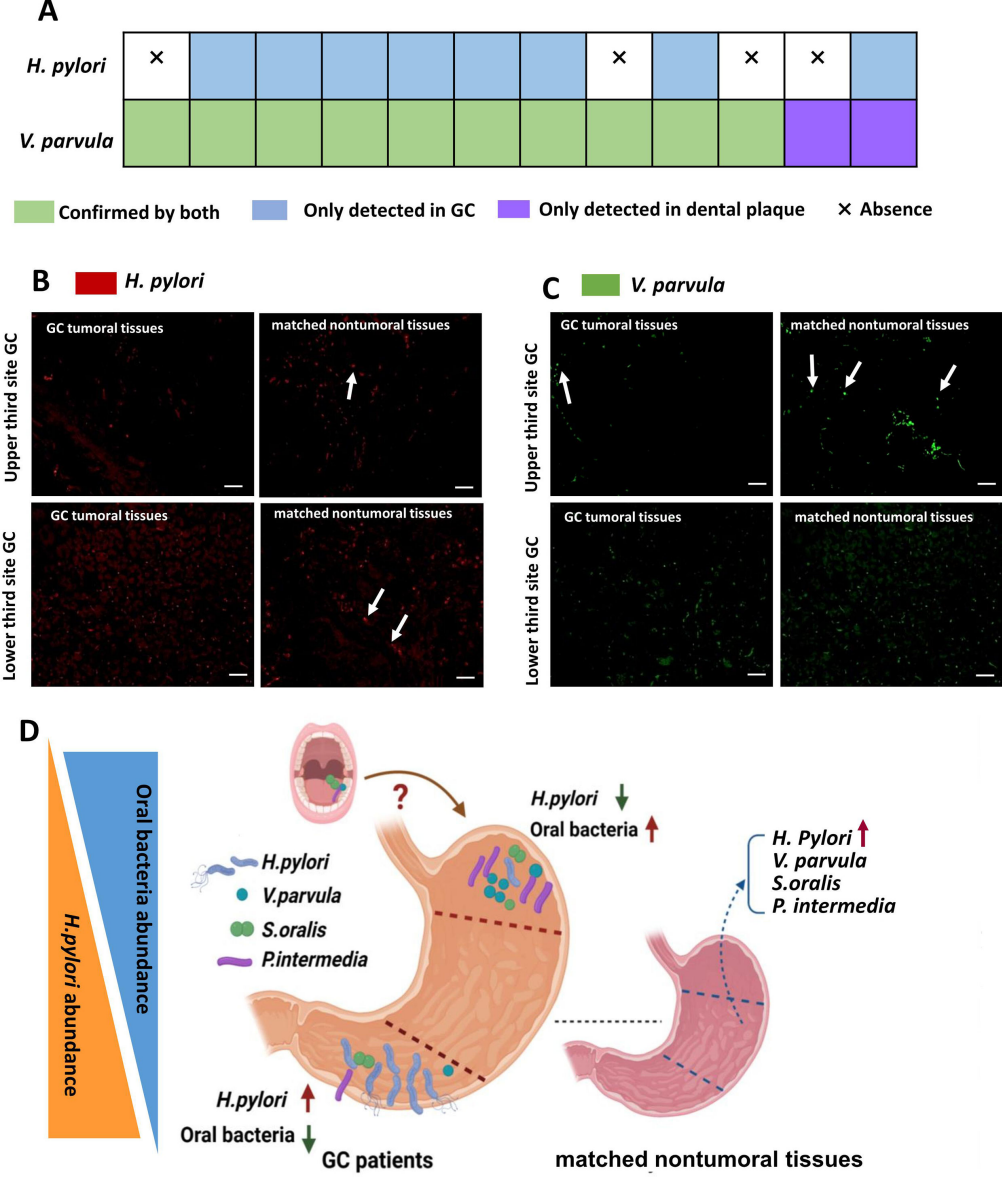
The presence of an oral microbe in GC tissues harboring characteristic bacteria consistent with oral and gastric microbiome communities. (**A**) The presence/absence of *H. pylori* and *V. parvula* was validated in both GC tumoral tissues (*n* = 12) and paired dental plaque samples (*n* = 12) using the 16S amplicon sequencing method. (**B**) Representative FISH images for *H. pylori* (white arrows) in GC tumoral tissues showed distinct differences from matched nontumoral tissues, scale bar = 50 µm. (**C**) Representative FISH images for *V. parvula* (white arrows) in GC tumoral tissues and matched nontumoral tissues, scale bar = 50 µm. (**D**) The oral-associated species were observed in the upper third GC.

We found that *V. parvula* and *S. oralis* were the most enriched species in the GC tumoral tissues compared to the matched nontumoral tissue in differential abundance expression (Table S5). To test whether the core oral-associated gastric microbiota was observed in dental plaque and GC tissue samples, we next challenged the core oral-associated gastric microbiota in independent validation cohorts. We further verified the prevalence of *H. pylori* and *V. parvula* by third-generation sequencing. Generally, the relative abundances of the top 10 bacterial species in both the dental plaque and GC tumoral tissues included *V. parvula* (Table S6), which was concordant. Intriguingly, *H. pylori* was not detected in any of the dental plaque samples (Table S7), even though it was detected by sequencing GC tissue samples from 8/12 subjects (Fig. 6A; Fig. S5). In a subset of four GC patients with tumoral tissues and matched nontumoral tissues, we further verified the prevalence of *H. pylori* (Fig. 6B) and *V. parvula* (Fig. 6C) using FISH assays with specific labeled probes. The results showed the *in situ* DNA of *H. pylori* and *V. parvula* in the upper and lower sites of the stomach, both in tumoral and matched nontumoral tissues. *H. pylori* was more abundant in the matched nontumoral tissue of the upper and lower sites (especially the lower site) than in the tumoral tissue. *V. parvula* was more obvious in the gastric upper site. Generally, the amplicon and FISH data were concordant with the 16S rRNA amplicon-based analyses.

## DISCUSSION

In the present study, we observed significant spatial heterogeneity of microbial diversity in GC tumoral tissues and matched nontumoral tissues. We found that the vertical distribution of the gastric microbiota was likely an important factor causing microbial diversity in GC tumoral tissues. The co-occurrence of the oral-associated microbiota cluster, including *V. parvula*, *S. oralis*, and *P. intermedia*, was enriched in the upper third of the GC, whereas *H. pylori* was more abundant in the lower third site. We adopted the co-occurrence and co-exclusion patterns of *H. pylori*, the *Pseudomonas* module, and the oral-associated microbiota module. This indicated that the oral-associated microbiota module was negatively correlated with the *Pseudomonas* module in the upper third site of the GC but positively correlated with the *Pseudomonas* module in the lower third site of the GC. In addition, *H. pylori*-negative GC patients with oral-associated gastric microbiota had worse overall survival, while the increase in microbial abundance in *H. pylori*-positive GC patients showed no difference in overall survival. Our findings indicate that the oral taxon is clinically significant as an independent prognostic factor for GC in overall survival analysis, especially in *H. pylori*-negative GC patients.

Consistent with an earlier study (27, 28), our study revealed that *Helicobacter* is the foundational taxon of the consortium of tumoral tissues and matched nontumoral tissues. Interestingly, the abundance of *H. pylori* was highest in matched nontumoral tissue, followed by peritumoral tissue, and lowest in GC tissue. Notably, there are conflicting views regarding the role of *H. pylori* in the pathogenesis of gastroduodenal diseases (29). The prevalence of *H. pylori*-negative gastritis is approximately 21% in the United States (30). A possible explanation for the enrichment of *H. pylori* in matched nontumoral tissues may be the decreased acid secretion, and *Helicobacter* plays a role in the degradation of amino sugar and nucleotide sugar metabolites (28). In the present study, we found that the alpha diversity of the gastric microbiota was significantly greater in the matched nontumoral tissues of *H. pylori*-negative patients than in those of *H. pylori*-positive patients in the upper third. However, further studies are needed to confirm the roles of *H. pylori*-associated microbiota constituents from matched nontumoral tissues in the pathogenesis of GC. In a recent publication, gastric juice samples were collected from GC patients, and the most abundant bacteria were *Streptococcus*, *Prevotella*, *Pseudomonas*, and *Helicobacter* (31). A meta-analysis revealed that oral microbes were significantly enriched in GC compared to the precancerous stages (32). In the present study, the abundances of genera, including *Pseudomonas*, *Streptococcus*, and *Sphingomonas*, were increased in GC tumoral tissues. Notably, *S. anginosus* is enriched in the gastric mucosa of GC patients and promotes gastric tumorigenesis via direct interactions with gastric epithelial cells (33).

Historically, the stomach of healthy individuals has been considered a sterile organ, as the acidic conditions of the stomach are unfavorable for bacterial colonization (34). Previous studies have identified the main bacterial components of the gastric microbiota, including *Proteobacteria*, *Firmicutes*, *Bacteroidetes*, *Fusobacteria*, and *Actinobacteria,* in healthy subjects (35, 36). However, these healthy gastric tissues, which have an uneven distribution of lesions, are limited by the use of samples from endoscopic biopsies. In the present study, we isolated the entire mucosa-adherent and intraepithelial bacterial sample DNA for analysis, which indicated that *H. pylori* comprises more than one-third of the GC microbiota. The connectivity and robustness of the microbiota co-occurrence networks increased in the upper and lower third of the sites, presenting a mode of “high–low–higher” (Fig. 4). Poorly developed microbial networks usually have low functionality in supporting microbial function in maintaining ecosystems (37, 38). Both CGC and NCGC occur in adjacent organs and together account for approximately 90% of GC cases (17). Thus, we speculate that a greater degree of closeness of the microbiota network in the upper and lower third of the network contributes to gastric carcinogenesis. Interestingly, the alpha diversity of the gastric microbiota significantly increased at the upper third site, with a small individual variance. This finding indicates that the microbial community in the upper third site has greater environmental stress and greater connectivity with the gastric microbiota network, especially during CGC tumorigenesis.

Notably, we observed that *H. pylori* was more abundant in the lower third site of GC, whereas oral bacterial taxa were enriched in the upper third site of GC. Chronic *H. pylori* infection is the main cause of non-CGCs (11). These findings indicate a reduced risk of *H. pylori* infection associated with CGC. This tendency is consistent with studies revealing a reduced risk of CGC associated with *H. pylori* infection (16). There has been an alarming increase in the incidence of CGC, while that of NCGC has decreased in recent decades (17). Moreover, CGC is often identified in advanced stages, indicating a poor prognosis (39). Thus, understanding the risk and role of gastric microbial components other than *H. pylori* in the etiology of CGC is important for risk reduction. Consistent with the co-exclusion relationship of *Pseudomonas* and *H. pylori* in the present study, *Pseudomonas* was reportedly decreased in the gastric microbial community of *H. pylori*-positive individuals but was dominant in *H. pylori*-negative individuals (40). Although the effects of the core oral-associated gastric microbiota require further investigation, the keystone module determined by the microbial network may be a new prognostic indicator. We used the overall survival rate as a prognostic indicator, which estimates the chance of remaining alive at a certain time after diagnosis (41). Additionally, the overall survival rate is widely used and is an easily understood survival measure (42, 43). In the present study, the overall survival rate was the endpoint of death from all causes and mostly included GC-related deaths.

For *H. pylori*-negative GCs, lower oral-associated microbial diversity indicated worse 5-year survival when adjuvant chemotherapy was administered (Fig. 5K). Ectopic colonization of gastrointestinal oral flora may cause dysbiosis in patients with gastroduodenal diseases (29). We explored the roles of oral-associated species (44, 45), including *V. parvula*, *S. oralis*, and *P. intermedia* in *H. pylori*-negative GCs and their implications for prognosis. Patients with GCs harboring oral microbial taxa had worse overall survival and could benefit less from chemotherapy. Notably, *V. parvula*, *S. oralis*, and *P. intermedia* were the most enriched species in the GC tumoral tissues compared to the matched nontumoral tissues in terms of differential abundance (Table S5). We further performed Kaplan‒Meier analyses, which indicated that *V. parvula* and *S. oralis* were negatively associated with overall survival in GC patients (Fig. S6), indicating future directions for the use of bacterial markers for predicting overall survival and chemotherapy sensitivity (46).

Recently, the interaction and relationship between *H. pylori* and the oral microbiota in GC carcinogenesis have been discussed, and further evidence of the mechanisms underlying oral microbiota communities is needed for the identification of new prevention methods for GC (21). He et al. also reported that *Veillonella* was identified in both the gastric mucosa and gastric fluid of GC patients and was further validated in an independent cohort (47). *V. parvula*, as an important component of oral microecology, regulates neutrophils during the immune response and releases reactive oxygen species (48). As a “bridging bacterium,” *V. parvula* contributes to early colonization and mediates subsequent bacterial aggregation (49). The predominant *Veillonella* sp. (50) is resistant to acidic environments. *P. intermedia* is a periodontal pathogen that plays an important role in the formation of dysbiosis biofilms during the development of periodontitis (51). A recent study indicated that elevated bile acid levels were positively associated with lipopolysaccharide-producing bacteria such as *P. melaninogenica* in patients with bile reflux gastritis and GC (52). In independent validation cohorts, we detected that the prevalence of *V. parvula* in both dental plaque (12/12 subjects) and GC tissue samples (10/12 subjects) was concordant. Intriguingly, the abundance of *V. parvula* in dental plaque samples from GC patients is much greater than that in a healthy population (53). In addition, the relative abundance of *Fusobacterium* spp. was greater in GC tumoral tissues than in normal tissues (Table S2). Recent studies have reported that *Fusobacterium* is associated with GC. An increased abundance of *Fusobacterium* in the gastric microbiota of GC patients is associated with mutations and the progression of GC (54).

However, our study has several limitations. The degree to which this association is a consequence of oral-associated bacterial enrichment transiting through the gut (55) or because oral bacteria can directly drive GC tumorigenesis at distal sites remains debatable. *H. pylori* infection can cause alterations in proteins normally localized to the gastric corpus, which might be associated with the development of GC (56, 57). Whether a 1% cutoff (10, 58) is sufficient for *H. pylori* in dental plaque or other bacterial species has not yet been tested in the present study, which is also an important direction for future research. In addition, further metagenomic sequencing could provide significant functional profiles between oral bacteria and *H. pylori* (59).

### Conclusions

In conclusion, we described the microbiota-scale biogeography of GC and found that gastric vertical distribution was likely an important factor causing microbial diversity in GC tumoral tissues. The co-occurrence of the oral-associated microbiota cluster was enriched in the upper third site of the GC, whereas *H. pylori* was more abundant in the lower third site. For the gastric ecosystem, the co-occurrence patterns indicated that the oral-associated microbiota module was negatively correlated with the *Pseudomonas* module in the upper third site of the GC but positively correlated with the *Pseudomonas* module in the lower third site of the GC. In addition, *H. pylori*-negative GC patients with oral-associated gastric microbiota showed worse overall survival. We showed that oral-associated species, including *V. parvula* and *S. oralis*, were correlated with overall survival. Thus, our study highlights the roles of the oral-associated microbiota in the upper third of the GC. In addition, oral-associated species may serve as noninvasive screening tools for the management of GC, concentrating more on oral hygiene, and may be an independent prognostic factor for *H. pylori*-negative GCs.

## MATERIALS AND METHODS

### Study design and patient’s cohort

The samples and clinical information used in this study were obtained under the conditions of informed consent and with the approval of the institutional review board of West China Hospital, No. 2021(1430). The data used in this study were obtained from Chinese adults. Participant records were anonymized and de-identified, and signed informed consent forms were obtained prior to the analysis. The follow-up was conducted from April 2013 to October 2021. A total of 223 GC patients who underwent gastrectomy were recruited from four clinical service centers (West China Hospital headquarters, Wenjiang branch clinical center, Shangjin branch clinical center, and Tianfu branch clinical center). The diagnosis of primary GC in all participants was confirmed using upper gastrointestinal endoscopy and biopsy. Participants with primary GC were included under the following conditions: (i) they were clearly diagnosed with primary gastric cancer before surgery; (ii) pathological examination confirmed that they had received R0 resection and curative resection with negative residual margins. Participants were excluded if they had any of the following situations: (i) administration of antibiotics for *H. pylori* during the 1 year prior to surgery; (ii) any preoperative chemotherapy or radiotherapy; (iii) multiple stomach tumors; (iv) another malignancy or any other life-threatening disease diagnosed during the 3 years prior to the operation; (v) incomplete clinicopathological information or loss of follow-up; or (vi) death due to postoperative complications in the hospital (see Table S1). Clinicopathological characteristics, including gender, age, tumor cross-sectional and vertical location, body mass index, macroscopic type, Lauren type, tumor differentiation, *H. pylori* status, *Her* 2 status, tumor size, T stage, N stage, M stage, TNM stage evaluated according to the 7th edition of the AJCC TNM staging system, and follow-up information were collected.

A total of 223 patients with GC were enrolled in this study. In total, 223 GC tumoral tissues and corresponding relevant matched nontumoral tissues were compared. Of the 223 GC participants, peritumoral tissues were included in the additional analysis. The peritumoral tissues (2 cm) were adjacent to the GC tumoral tissues, and matched nontumoral tissues were more than 5 cm adjacent to the GC tumoral tissues. Samples were collected from 223 participants with primary GC who underwent radical gastrectomy and were preserved in the West China Hospital Biobank for microbiota analysis. After gastrectomy, the samples were rinsed with sterile phosphate buffered saline (PBS), placed directly on dry ice, and stored in liquid nitrogen (28) for 16S rRNA gene profiling using third-generation sequencing.

After curative resection of GC, participants were periodically followed-up by letters, telephone interviews, and outpatient visits. The follow-up was performed every 3 months during the first 2 postoperative years and every 6 months after the second year. All surviving patients were followed up annually until death. Survival time was defined as the time from the date of surgery to the last contact time (October 2021) or the date of death. Gastric samples with ≤1% *H. pylori* relative abundance were defined as *H. pylori*-negative, while gastric samples with >1% *H. pylori* relative abundance were *H. pylori*-positive, as previously described (10). Similarly, gastric samples with a ≤1% relative abundance of species were regarded as negative. With the optimal OTU cut-off value of 207 determined by X-tile using the minimum *P* value from the log-rank *χ*^2^ test, samples were classified into high and low microbial diversity groups.

An independent validation population (*n* = 12) was recruited from the Gastric Cancer Center and Laboratory of Gastric Cancer from January 2022 to June 2022 using the same protocols for biological sample collection, processing, and storage. Matched dental plaque samples were collected from the buccal surfaces of the anterior teeth and first mandibular molar using sterile dental probes and pooled. The pooled samples were kept in sterilized tubes containing PBS buffer on ice, transferred to the laboratory within 2 h, and stored at −80°C immediately until DNA extraction.

### DNA extraction and sample sequencing

The frozen tissues were soaked in DNA/RNA Shield Reagent (ZYMO Research) prior to DNA extraction. The dissolved tissues were mixed with Solid Tissue Buffer from a Quick-DNA Miniprep Plus Kit (D4069, ZYMO Research), digested with Proteinase K, and disrupted by beads beating (ZR BashingBead Lysis Tube) as previously described (33). The dental plaque samples were mixed with BioFluid and Cell Buffer from a Quick-DNA Miniprep Plus Kit (D4069, ZYMO Research). Then, the bacterial genomic DNA was extracted from gastric tissues, and dental plaque samples followed the Quick-DNA Miniprep Plus Kit manufacturer’s protocols. The extracted DNA was amplified using universal bacterial primers 8F (5’- AGAGTTTGATCATGGCTCAG-3′) and 1492R (5’- CGGTTACCTTGTTACGACTT-3′) to amplify the full-length 16S rRNA gene. To avoid errors during cluster identification due to the high similarity of bases across all amplicons, we tagged a 24 bp barcode with forward primers. The 20 µL PCR volume contained 10 ng of extracted DNA and 0.3 µM primers with Kodaq 2 × PCR MasterMix (G497, Applied Biological Materials Inc.). PCR was conducted with an activation step at 95°C for 3 min, followed by 10 touchdown cycles at 95°C for 10 s, 65°C (decrease of 1°C for each cycle) for 20 s, and 68°C for 90 s, followed by 20 cycles at 95°C for 10 s, 68°C for 90 s, and a final elongation for 7 min. Amplifications were quantified using a Qubit dsDNA HS Assay Kit (Q32854, Thermo Fisher Scientific) on a Tecan F200 luminescent plate reader, and samples were pooled to equimolar amounts of DNA. Three PCR replications were performed for each sample, and sequencing was performed after equal mixing. TE buffer was added as a negative control. The library was prepared as recommended by Nanopore (SQK-LSK109, Oxford Nanopore Technologies, ONT) and loaded onto a GridION ×5 flow cell (FLO-MIN106 R9.4.1, Oxford Nanopore Technologies) for a 72-hour sequencing protocol using MinKNOW software at Rhonin BioSciences Company (Chengdu, China). The sequencing reads were deposited in National Center of Biotechnology Information (NCBI) SRA and the accession number (PRJNA925240). Herein, we set up the bacterial microbiota information of human GC in an online publicly available database, namely the Huaxi Gastric Cancer Microbiome Database, which is accessible at http://www.hgcmd.cn/.

### Bioinformatics and statistical analyses

Base calling of FAST5 format ONT sequencing data was performed using Guppy v4.2.2. Demultiplexing and barcode trimming of the FASTA format base called data were conducted using qcat v1.1.0. Data quality control was performed using the NanoFilt v2.7.1. Sequences with lengths less than 1,300 bp or greater than 1,600 bp and an average quality of less than 10 were removed. The taxonomy of each sequence was annotated using Kraken2, based on the SILVA database v138. A community feature table (OTU table-like) was generated using a homemade R script by integrating the sequence number at the species level. The scripts used for the analysis are available on the associated public website (https://github.com/vesperlight2/Kraken2OTUtable). The singleton of each taxon was removed to avoid false positive results. All OTUs in each sample were randomly resampled to the minimum number of OTUs in all samples. All statistical analyses were performed using R v4.1.0 (https://www.R-project.org/). Alpha diversity was calculated using the vegan R package. Differences in alpha diversity were assessed using the Kruskal–Wallis rank-sum test for paired groups.

To investigate the effect of spatial differences or *H. pylori* infection on bacterial community stability, beta diversity analysis was performed. To further explore the co-occurrence and co-exclusion patterns of prokaryotic communities, we performed Spearman’s rank correlations on the relative abundance of OTUs in all groups (*P* < 0.001 |*r*| > 0.6). The network was constructed, visualized, and summarized by Gephi (v 0.9.2) and R package. Differential taxa analysis between groups was performed using random forest analysis. Random forest analysis was performed using the R package, random forest. The overall survival curves were assessed using the R package survival.

### FISH microscopy

For peptide nucleic acid (PNA), GC tumoral tissues and matched nontumoral tissues (*n* = 4) were obtained immediately after surgical excision. Briefly, the tissues were fixed in freshly prepared 4% paraformaldehyde (60). Tissue sections of 5 µm thickness were mounted and rehydrated using a graded ethanol series (100%, 95%, 80%, 70%, and 50%) for 5 min each time and permeated with proteinase K (Wuhan Servicebio Technology Co., Ltd.). Finally, all smears were washed with distilled water for 10 min and allowed to air-dry. PNA-FISH was conducted using a GenePharma FISH Kit (GenePharma Technology Co., LTD, Shanghai, China) according to the manufacturer’s instructions. For prehybridization, 100 µL of prehybridization solution without probe was applied for 8 min at 78°C. From the genomic DNA, a Cy3 -labeled PNA probe (5′-CACACCTGACTGACTATCCCG-3′) targeting *H. pylori* 16S rRNA and an Fluorescein Isothiocyanate (FITC)-labeled PNA probe (5′-AGACGCAATCCCCTCCTT-3′) targeting *V. parvula* 16S rRNA (Wuhan Servicebio Technology Co., Ltd.) were used. The slides were then incubated with a hybridization solution containing 200 nM of the PNA probe overnight at 37°C. Subsequently, the slides were washed twice with PBS and air-dried. The slides were further examined using a fluorescent microscope at 40× magnification. The completed Strengthening the Reporting of Observational Studies in Epidemiology (STROBE) checklist to a public data repository with a link (https://zenodo.org/records/11397753).

## Data Availability

The sequencing reads were deposited in NCBI SRA and the accession number (PRJNA925240). The scripts used for the analysis are available on the associated public website (https://github.com/vesperlight2/Kraken2OTUtable). The completed STORMS checklist to a public data repository with a link (https://zenodo.org/records/11397753). Other data sets generated and/or analyzed during the current study are available from the corresponding author upon reasonable request.

## SUPPLEMENTAL MATERIAL

Fig. S1 msystems.00089-24-s0001.pdf Heat map showing the correlation between tumor tissue species flora and clinicopathological features.

Fig. S2 msystems.00089-24-s0002.pdf Heat map for the top 20 abundant microbial species in GC, peritumoral tissues, and matched nontumoral tissues.

Table S1 msystems.00089-24-s0003.xlsx Clinicopathological characteristics of 223 participants.

Table S2 msystems.00089-24-s0004.xlsx Gastric microbiota summary of GC, peritumoral tissues, and matched nontumoral tissues.

Table S3 msystems.00089-24-s0005.xlsx Alpha diversities of different between *H. pylori*-positive and *H. pylori*-negative individuals.

Table S4 msystems.00089-24-s0006.xlsx Permutation test based on the Bray-Curtis distance between *H. pylori*-positive and *H. pylori*-negative individuals.

Table S5 msystems.00089-24-s0007.docx Differential abundance in GC tumoral and matched nontumoral tissues.

Additional supplemental figures msystems.00089-24-s0008.pdf Fig. S3–S6.

Additional supplemental tables msystems.00089-24-s0009.xlsx Tables S6 and S7.

Legends msystems.00089-24-s0010.docx Supplemental legends.

ASM does not own the copyrights to Supplemental Material that may be linked to, or accessed through, an article. The authors have granted ASM a non-exclusive, world-wide license to publish the Supplemental Material files. Please contact the corresponding author directly for reuse.
