# Supplementary figures and images for: Distinct oral-associated gastric microbiota and Helicobacter pylori communities for spatial microbial heterogeneity in gastric cancer
Source: mSystems. 2024 Jun 28;9(7):e00089-24. doi: 10.1128/msystems.00089-24 (PMC11265414; doi:10.1128/msystems.00089-24)

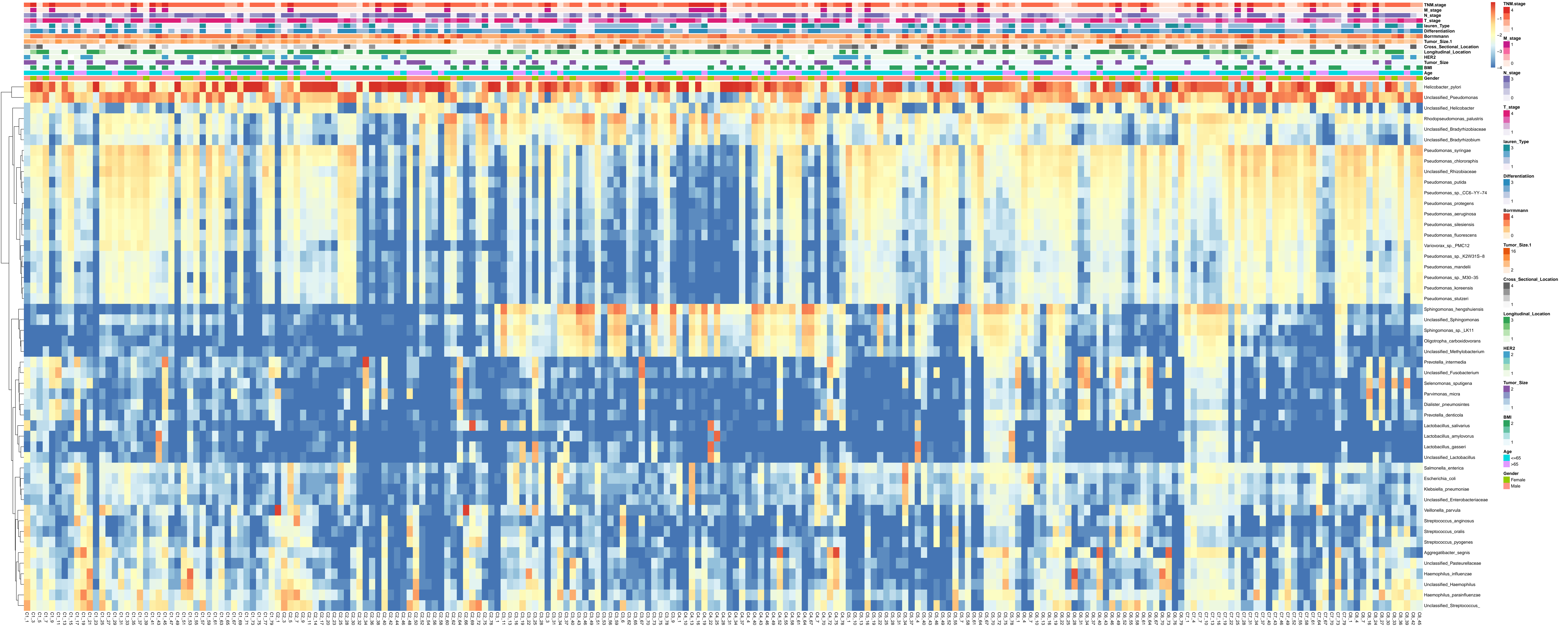

Supplement: Fig. S1 — Heat map showing the correlation between tumor tissue species flora and clinicopathological features. [file msystems.00089-24-s0001.pdf]
