# Supplementary material for: Distinct oral-associated gastric microbiota and Helicobacter pylori communities for spatial microbial heterogeneity in gastric cancer
Source: mSystems. 2024 Jun 28;9(7):e00089-24. doi: 10.1128/msystems.00089-24 (PMC11265414; doi:10.1128/msystems.00089-24)
Supplement: Table S5 — Differential abundance in GC tumoral and matched nontumoral tissues. [file msystems.00089-24-s0007.docx]

| **Bacterial species** | **C/N ratio** | **p value** | **Mean_C** | **Mean_N** | **SD_C** | **SD_N** |
| --- | --- | --- | --- | --- | --- | --- |
| *Selenomonas sputigena* | 4.183862 | 0.013654 | 0.68137 | 0.162857 | 2.440351 | 0.743598 |
| *Veillonella parvula* | 3.975666 | 0.009847 | 1.114506 | 0.280332 | 6.98643 | 0.739862 |
| *Streptococcus oralis* | 3.196809 | 0.003406 | 0.3884 | 0.121496 | 1.222029 | 0.635472 |
| *Prevotella melaninogenica* | 2.280685 | 0.007663 | 0.17212 | 0.075468 | 0.581661 | 0.201403 |
| *Gemella morbillorum* | 2.0837 | 0.000746 | 0.203786 | 0.0978 | 0.919967 | 0.270347 |
| *Dialister pneumosintes* | 2.022472 | 0.008581 | 0.284353 | 0.140597 | 1.053384 | 0.794109 |
| *Streptococcus anginosus* | 2.001635 | 0.000604 | 0.351564 | 0.175638 | 1.446675 | 0.711541 |
| *Streptococcus pyogenes* | 1.985915 | 0.002867 | 0.283491 | 0.142751 | 0.668102 | 0.347035 |
| *Streptococcus infantarius* | 1.789678 | 0.040228 | 0.231575 | 0.129395 | 0.533884 | 0.308298 |
| *Bacteroides salanitronis* | 1.785714 | 0.000319 | 0.154384 | 0.086455 | 1.78947 | 1.087355 |
| *Prevotella intermedia* | 1.587872 | 0.04373 | 0.8499 | 0.535244 | 4.759005 | 2.543685 |
| *Ralstonia pickettii* | 1.583846 | 0.005083 | 0.147849 | 0.093348 | 0.770924 | 0.597921 |
| *Aggregatibacter segnis* | 1.299837 | 0.028352 | 1.428515 | 1.098996 | 5.907449 | 4.770333 |
| *Clostridioides difficile* | 1.277684 | 0.013815 | 0.163216 | 0.127743 | 0.562693 | 0.563485 |
| *Stenotrophomonas maltophilia* | 1.26747 | 0.033529 | 0.11331 | 0.089399 | 0.127975 | 0.103876 |
| *Rhodopseudomonas palustris* | 1.14803 | 0.004558 | 2.561116 | 2.230879 | 4.464205 | 4.705479 |
| *Parvimonas micra* | 1.127617 | 0.012816 | 0.286148 | 0.253764 | 1.364308 | 1.822469 |
| *Helicobacter pylori* | 0.717298 | 0.000101 | 35.13742 | 48.98577 | 35.23659 | 37.68645 |

**Table S5. Differential abundance in** **GC tumoral and** **matched non-tumoral tissues**

C: GC tumoral tissues;

N: Matched non-tumoral tissues
