# Supplementary material for: Distinct oral-associated gastric microbiota and Helicobacter pylori communities for spatial microbial heterogeneity in gastric cancer
Source: mSystems. 2024 Jun 28;9(7):e00089-24. doi: 10.1128/msystems.00089-24 (PMC11265414; doi:10.1128/msystems.00089-24)
Supplement: Additional supplemental figures — Fig. S3–S6. [file msystems.00089-24-s0008.pdf]

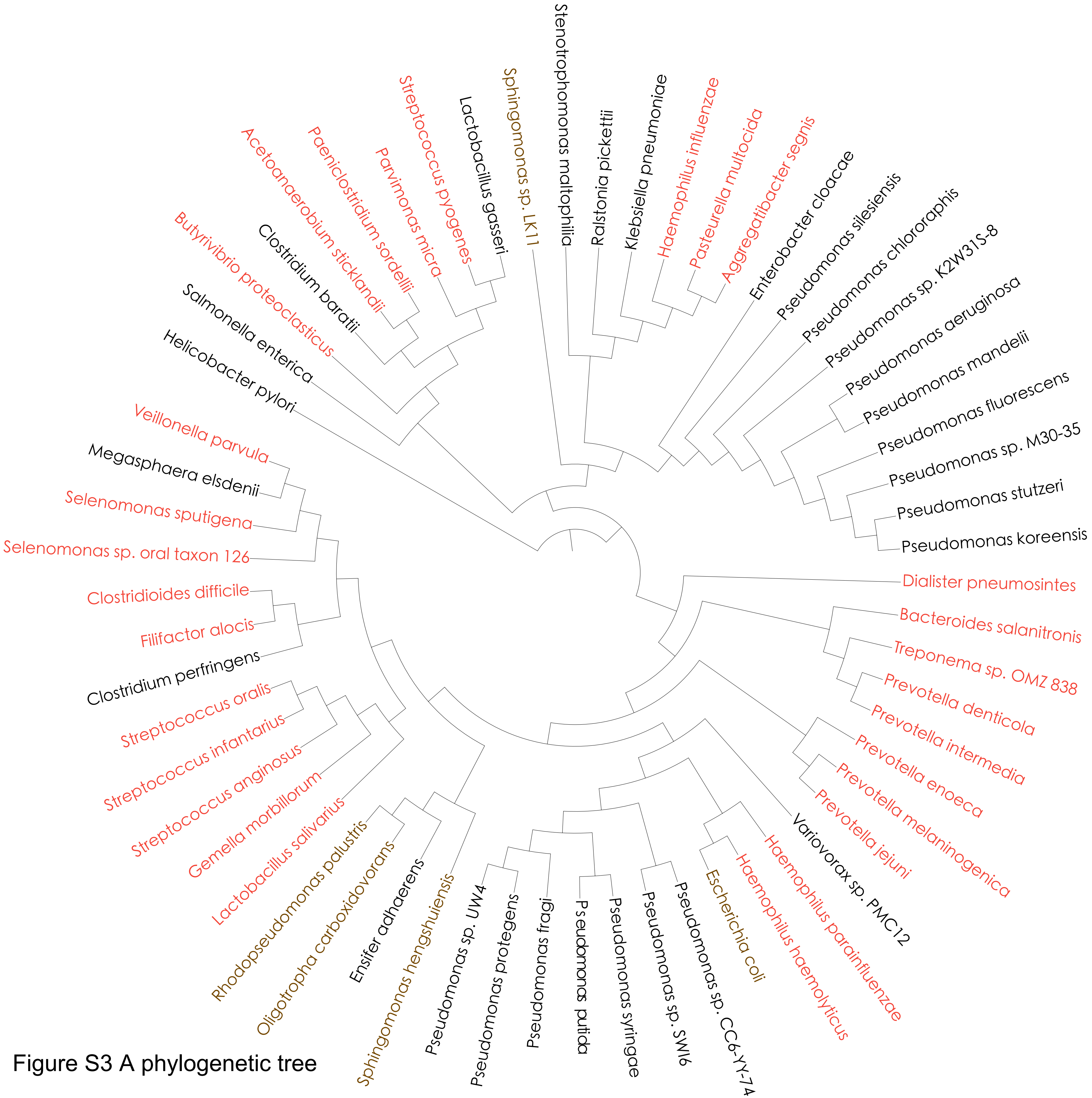

Figure S3 A phylogenetic tree

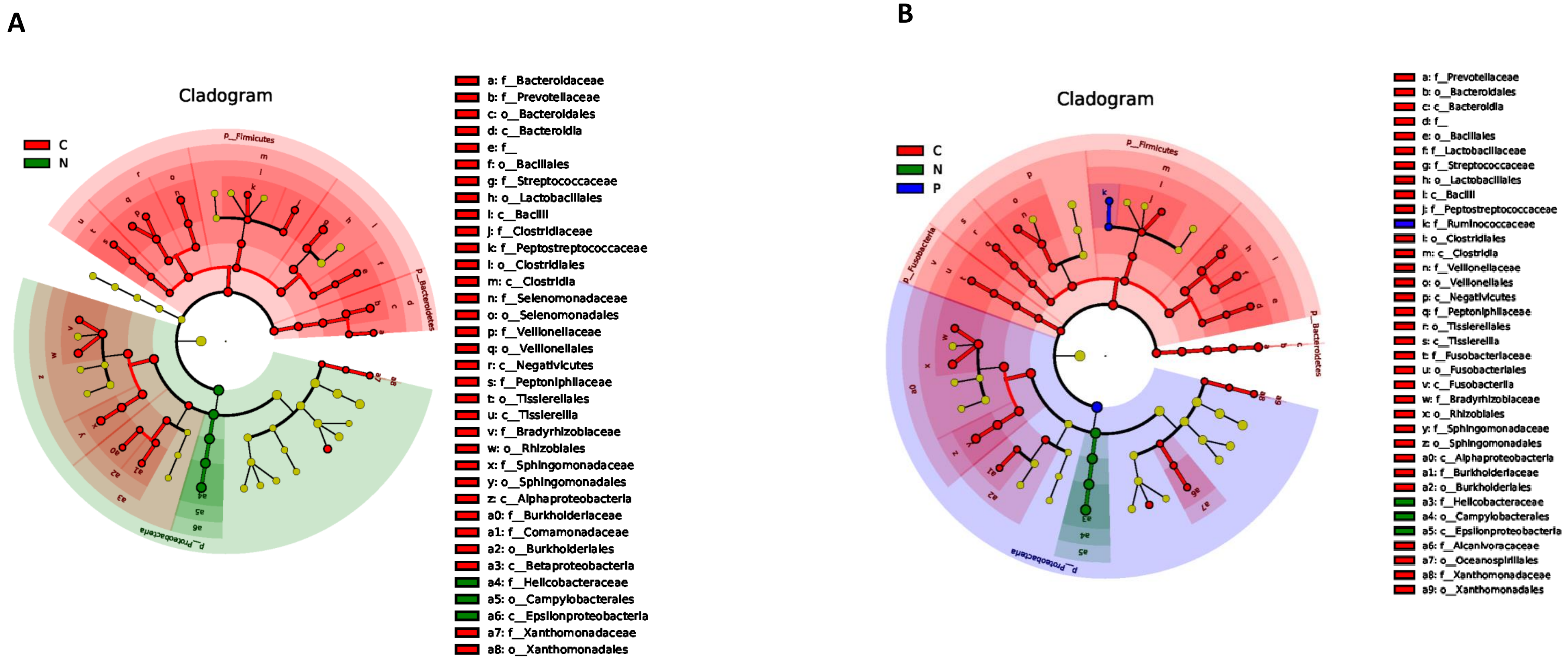

Figure S4 The LEfse analysis for GC, peritumoral tissues and matched non-tumoral tissues.

(A) LEfse analysis between GC and matched non-tumoral tissues.

(B) LEfse analysis for GC tumoral tissues, peritumoral tissues, and matched non-tumoral tissues.

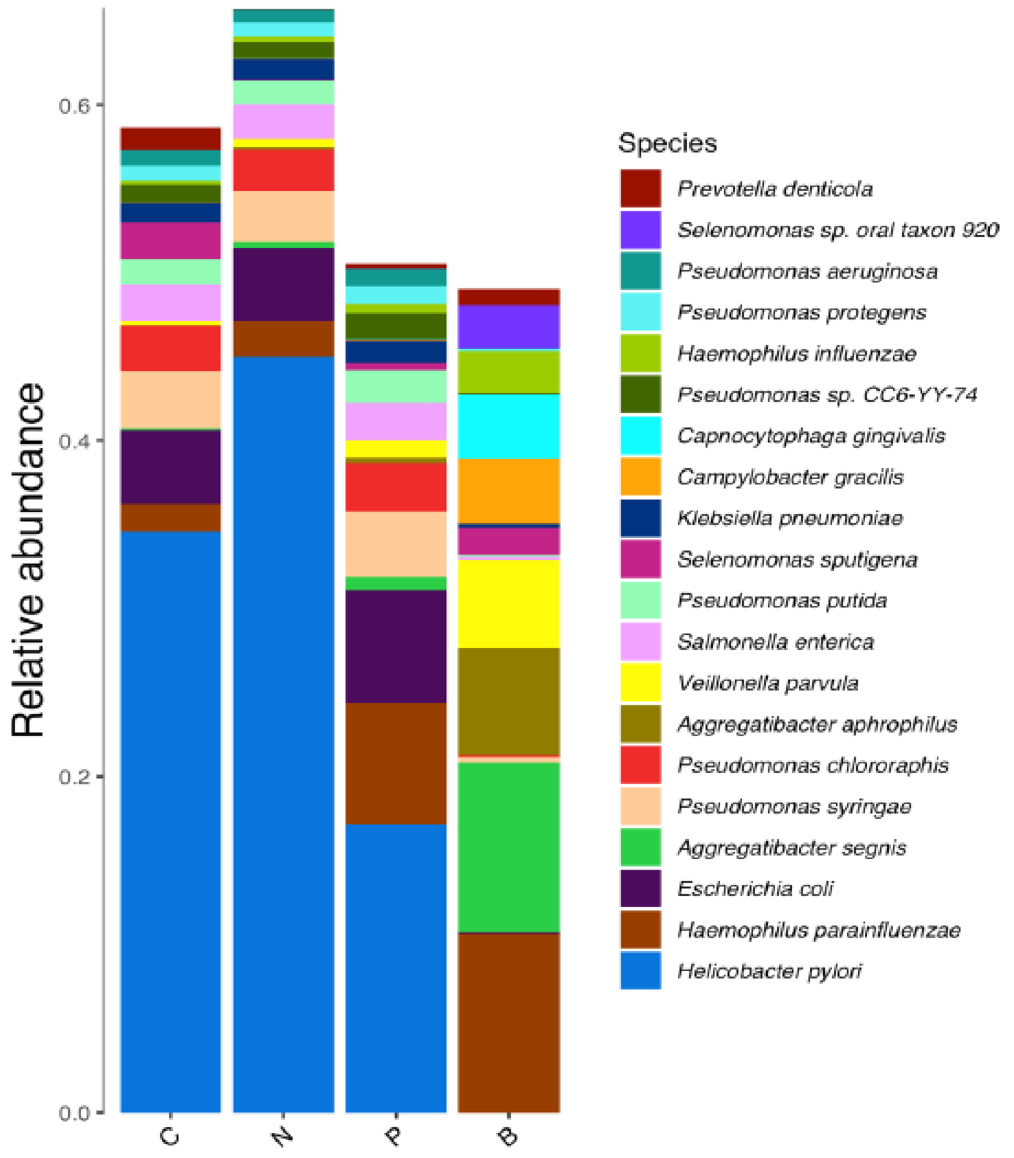

Fig.S5 Different bacterial community structures among for GC (C), peritumoral tissues (P), matched nontumoral tissues (N) and dental plaque samples (B) in an independent validation population

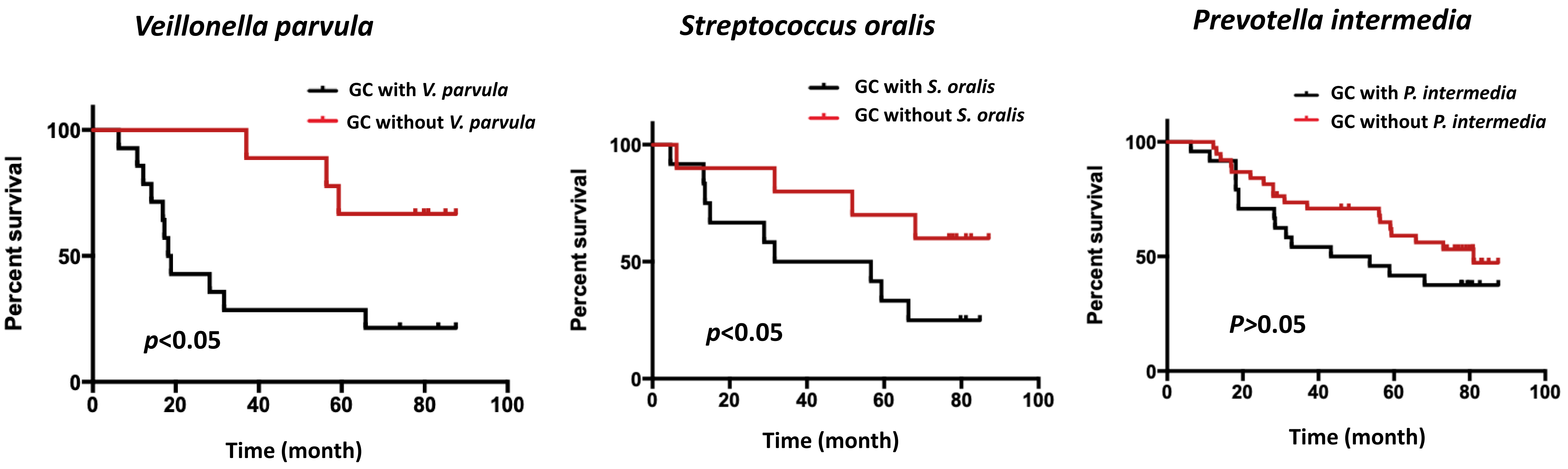

Fig.S6 Specific oral-associated species are negatively associated with the overall survival
