# Supplementary material for: Distinct oral-associated gastric microbiota and Helicobacter pylori communities for spatial microbial heterogeneity in gastric cancer
Source: mSystems. 2024 Jun 28;9(7):e00089-24. doi: 10.1128/msystems.00089-24 (PMC11265414; doi:10.1128/msystems.00089-24)
Supplement: Legends — Supplemental legends. [file msystems.00089-24-s0010.docx]

**Distinct oral-associated gastric microbiota and *Helicobacter pylori* communities for spatial microbial heterogeneity in gastric cancer**

**Short title: Spatial microbial heterogeneity in gastric cancer**

Lei Lei^1,2#^, Lin-Yong Zhao^2#^, Ran Cheng^1#^, Hongyu Zhang^1^, Mengying Xia^1^, Xiao-Long Chen^2^, Valentin Kudriashov^2^, Kai Liu ^2^, Wei-Han Zhang^2^, Han Jiang^1^, Yi Chen^3^, Liang Zhu^4,5^, Hongmei Zhou^1^, Kun Yang ^2^, Tao Hu^1*^, Jian-Kun Hu^2*^

^1^ State Key Laboratory of Oral Diseases & National Center for Stomatology & National Clinical Research Center for Oral Diseases & Frontier Innovation Center for Dental Medicine Plus, West China Hospital of Stomatology, Sichuan University

^2^ Gastric Cancer Center and Laboratory of Gastric Cancer, State Key Laboratory of Biotherapy, West China Hospital, Sichuan University, and Collaborative Innovation Center for Biotherapy.

^3^ Department of Gastrointestinal Surgery, State Key Laboratory of Biotherapy, West China Hospital, West China Medical Center, Sichuan University, and Collaborative Innovation Center for Biotherapy.

^4^Target Discovery Institute, Centre for Medicines Discovery, Nuffield Department of Medicine, University of Oxford.

^5^Chinese Academy of Medical Sciences (CAMS), CAMS Oxford Institute (COI), Nuffield Department

**Supplementary files**

**Supplementary Table S1** The clinicopathological characteristics of 223 participants.

**Supplementary Table S2** The gastric microbiota summary of GC, peritumoral tissues, and matched nontumoral tissues.

**Supplementary Table S3** The alpha diversities of different between *H. pylori*-positive and *H. pylori*- negative individuals.

**Supplementary Table S4** The permutation test based on the Bray-Curtis distance between *H. pylori*-positive and *H. pylori*- negative individuals.

**Supplementary Table S5** Differential abundance in GC tumoral and matched non-tumoral tissues.

**Supplementary Table S6** The gastric microbiota summary of GC, peritumoral tissues, matched nontumoral tissues and dental plaque in an independent validation population.

**Supplementary Table S7** The detailed gastric microbiota of GC, peritumoral tissues, and matched nontumoral tissues, and dental plaque microbiota in an independent validation population.

**Supplementary Figure S1** The heat map showing the correlation between tumor tissue species flora and clinicopathological features.

**Supplementary Figure S2** The heat map for the top 20 abundant microbial species in GC, peritumoral tissues, and matched nontumoral tissues.

**Supplementary Figure S3** A phylogenetic tree was constructed for the 16S rRNA genes of the core microorganisms

**Supplementary Figure S4** The LEfSe analysis of gastric microbiota among GC tumoral tissues, peritumoral tissues, and matched non-tumoral tissues.

**Supplementary Figure S5** Different bacterial community structures among for GC, peritumoral tissues, matched nontumoral tissues and dental plaque samples in an independent validation population (n=12).

**Supplementary Figure S6** Specific oral-associated species are negatively associated with the overall survival
