# Supplementary material for: Distinct oral-associated gastric microbiota and Helicobacter pylori communities for spatial microbial heterogeneity in gastric cancer
Source: mSystems. 2024 Jun 28;9(7):e00089-24. doi: 10.1128/msystems.00089-24 (PMC11265414; doi:10.1128/msystems.00089-24)
Supplement: Fig. S2 — Heat map for the top 20 abundant microbial species in GC, peritumoral tissues, and matched nontumoral tissues. [file msystems.00089-24-s0002.pdf]

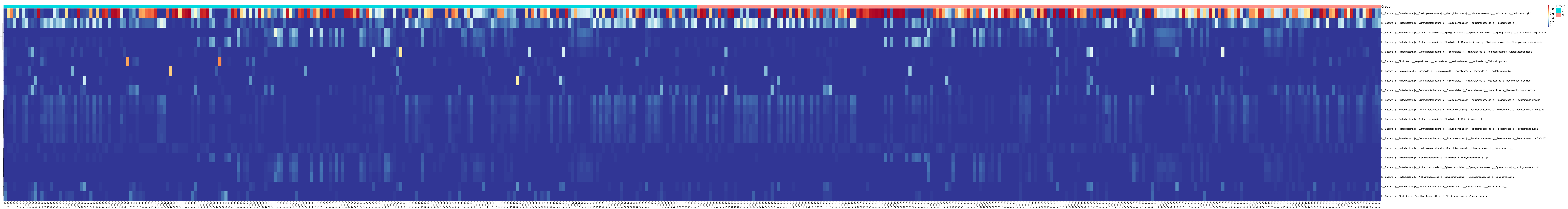
Group
k__Bacteria | p__Proteobacteria | c__Epsilonproteobacteria | o__Campylobacterales | f__Helicobacteraceae | g__Helicobacter | s__Helicobacter pylori
k__Bacteria | p__Proteobacteria | c__Gammaproteobacteria | o__Pseudomonadales | f__Pseudomonadaceae | g__Pseudomonas | s__
k__Bacteria | p__Proteobacteria | c__Alphaproteobacteria | o__Sphingomonadales | f__Sphingomonadaceae | g__Sphingomonas | s__Sphingomonas hengshuiensis
k__Bacteria | p__Proteobacteria | c__Alphaproteobacteria | o__Rhizobiales | f__Bradyrhizobiaceae | g__Rhodopseudomonas | s__Rhodopseudomonas palustris
k__Bacteria | p__Proteobacteria | c__Gammaproteobacteria | o__Pasteurellales | f__Pasteurellaceae | g__Aggregatibacter | s__Aggregatibacter segnis
k__Bacteria | p__Firmicutes | c__Negativicutes | o__Veillonellales | f__Veillonellaceae | g__Veillonella | s__Veillonella parvula
k__Bacteria | p__Bacteroidetes | c__Bacteroidia | o__Bacteroidales | f__Prevotellaceae | g__Prevotella | s__Prevotella intermedia
k__Bacteria | p__Proteobacteria | c__Gammaproteobacteria | o__Pasteurellales | f__Pasteurellaceae | g__Haemophilus | s__Haemophilus influenzae
k__Bacteria | p__Proteobacteria | c__Gammaproteobacteria | o__Pasteurellales | f__Pasteurellaceae | g__Haemophilus | s__Haemophilus parainfluenzae
k__Bacteria | p__Proteobacteria | c__Gammaproteobacteria | o__Pseudomonadales | f__Pseudomonadaceae | g__Pseudomonas | s__Pseudomonas syringae
k__Bacteria | p__Proteobacteria | c__Gammaproteobacteria | o__Pseudomonadales | f__Pseudomonadaceae | g__Pseudomonas | s__Pseudomonas chlororaphis
k__Bacteria | p__Proteobacteria | c__Alphaproteobacteria | o__Rhizobiales | f__Rhizobiaceae | g__ | s__
k__Bacteria | p__Proteobacteria | c__Gammaproteobacteria | o__Pseudomonadales | f__Pseudomonadaceae | g__Pseudomonas | s__Pseudomonas putida
k__Bacteria | p__Proteobacteria | c__Gammaproteobacteria | o__Pseudomonadales | f__Pseudomonadaceae | g__Pseudomonas | s__Pseudomonas sp. CC6-YY-74
k__Bacteria | p__Proteobacteria | c__Epsilonproteobacteria | o__Campylobacterales | f__Helicobacteraceae | g__Helicobacter | s__
k__Bacteria | p__Proteobacteria | c__Alphaproteobacteria | o__Rhizobiales | f__Bradyrhizobiaceae | g__ | s__
k__Bacteria | p__Proteobacteria | c__Alphaproteobacteria | o__Sphingomonadales | f__Sphingomonadaceae | g__Sphingomonas | s__Sphingomonas sp. LK11
k__Bacteria | p__Proteobacteria | c__Alphaproteobacteria | o__Sphingomonadales | f__Sphingomonadaceae | g__Sphingomonas | s__
k__Bacteria | p__Proteobacteria | c__Gammaproteobacteria | o__Pasteurellales | f__Pasteurellaceae | g__Haemophilus | s__
k__Bacteria | p__Firmicutes | c__Bacilli | o__Lactobacillales | f__Streptococcaceae | g__Streptococcus | s__
0.8
0.6
0.4
0.2
0
Group
C
N
